# Contrast CT Scans in the Emergency Department Do Not Increase Risk of Adverse Renal Outcomes

**DOI:** 10.5811/westjem.2016.4.28994

**Published:** 2016-06-29

**Authors:** Michael Heller, Paul Krieger, Douglas Finefrock, Thomas Nguyen, Saadia Akhtar

**Affiliations:** * Icahn School of Medicine, Department of Emergency Medicine, New York, New York; † Mount Sinai Beth Israel Medical Center, Department of Emergency Medicine, New York, New York; ‡ Hackensack University Medical Center, Department of Emergency Medicine, Hackensack, New Jersey

## INTRODUCTION

It has long been accepted that intravenous contrast used in both computed tomography (CT) and plain imaging carries a risk of nephropathy and renal failure, particularly in subpopulations thought to be at highest risk.^1^–^3^ Although early studies used high osmolality contrast media that is not typical of emergency department (ED) use today, the issue of contrast-induced nephropathy (CIN) is still an area of active interest with many studies appearing each year from many different specialties, on its pathogenesis, incidence, prevention and treatment.^4^–^7^ The plethora of data has usually focused on the incidence of CIN, usually defined as a small (such as 25% or an absolute increase of 0.5mg/dL) increase in creatinine after receiving intravenous (IV) contrast for either a particular indication (such as cardiac catheterization) or in a particular patient group (diabetics); the meaning of a creatinine rise in this setting is not at all clear, however.^8^–^10^ Many regimens have been proposed to ameliorate this creatinine rise, but there is a scarcity of data on what actual adverse clinical events occur and whether these can truly be ascribed to the IV contrast itself rather than the events that might well occur in a (usually) hospitalized population that required imaging. A few authors have even expressed doubt as to whether modern iodinated contrast (which is iso-osmolal) is a nephrotoxin.^11^–^13^

The primary objective of this retrospective, computerized chart review was to investigate an ED population of patients receiving IV contrast for CT scanning for the occurrence of two patient-oriented outcomes, death and dialysis, and compare this incidence to a contemporaneous control group of ED patients receiving similar CTs but without IV contrast. We also sought to determine if the incidence of CIN, as traditionally defined, was actually higher in the contrast group. Note that we use the traditional term “CIN” for those exhibiting a creatinine rise after CT scanning even though no patient in the control group actually received contrast.

## METHODS

The study patients were all adults seen in a six-year period in the ED of an active urban teaching hospital with a census between of approximately 75,000 starting in May 2005, and concluding in May 2010. The usual practice during that time was to use IV and oral contrast for all abdominal CTs unless the creatinine was greater than 1.5mg/dL or the study was for renal colic. Rare exceptions could occur in cases of major trauma (the ED is not a Level I trauma center) or unusual clinical circumstances. Chest CTs could be either with or without contrast depending on the indication; again contrast was not used with a creatinine greater than 1.5mg/dL. To be included in the study, the patient had to have been admitted to the hospital and have at least one ED creatinine (less than 1.6mg/dL) recorded and at least one additional serum creatinine measured in the subsequent 96 hours. There were no other inclusion or external criteria applied. No patient meeting these simple inclusion criteria were excluded. We also searched for the discharge condition of “death” and the procedure, “dialysis,” to identify two unambiguously relevant adverse patient-oriented outcomes. No patients were excluded if they fulfilled the inclusion criteria. The control group consisted of patients during that same period fulfilling the criteria for CT scanning, admission and creatinine testing but who received no IV contrast. All IV contrast material during the study period was non-ionic and the standard dose was 100mL per patient. Two different IV products were used during the six-year study period, Omnipaque 240 (GE Princeton, NJ) and Isovue 300 (Bracco, Italy), depending on which supplier was used at a given time. The decision as to which agent was available at any given time was dictated purely by cost considerations at the institutional purchasing level. The use of the two agents varied at least four times during the study period; Omnipaque 240 was used during the last three years of the study. Although oral contrast agents are not traditionally considered a significant risk in post-imaging creatinine elevation, the oral agent used from 2005 to 2008 was Gastrografin (Bracco, Italy) (20mL in 950mL of water). From 2008 through 2010 the oral agent used was Omnipaque 240 (25mL in 950mL of water), a diluted concentration of the same agent used as intravenous contrast during that time.

The study received a waiver for patient consent and an expedited approval from the institutional review board. We analyzed all data using Stata 11.0. Data on adverse events (death, dialysis) were compared using chi-square; creatinines were compared using students test; alpha was set at 0.05. A single investigator was responsible for building the dataset for both the contrast and control groups. The elements of the dataset, prior to de-identification, are enumerated in Table 1. The investigator was aware that the study’s purpose was to compare the incidence of CIN as traditionally defined in those ED patients who actually received IV contrast for a CT with those patients receiving a CT who did not receive contrast. We also compared the two patient-oriented outcomes of death and dialysis in the two groups. Although this investigator was not blinded to the study hypothesis, no charts were reviewed or abstracted as all patients fulfilling the inclusion criteria (two creatinine measurements in 96 hours and completion of an abdominal or chest CT) were included in the analysis.

## RESULTS

There were 6,954 patients in the contrast group vs. 909 patients in the non-contrast cohort. Every patient receiving an abdominal or chest CT during the six-year period fulfilling the admission criteria was included. The contrast and non-contrast groups did not differ in any parameter examined (Table 1). The age of both groups was nearly identical (both mean 54 years with std dev. 19.4 yr vs. 18.1 yr respectively). The contrast group was 57%/43% female to male compared with a 53%/47% ratio in the controls. Likewise, there was no significant difference in the incidence of diabetes in the two groups. For the primary outcomes of clinically significant adverse events, see Table 2. There were 106 deaths in the 6,954 patient contrast group versus 11 deaths in the 909 patient control group (1.5%, 95% CI [1.5%–1.8%] vs. 1.3%, 95% CI [0.7%–2.3%]; p=0.24). There were 16 patients in the contrast group (0.23%, 95% CI [0.1–0.4]) who required dialysis versus none in the non-contrast controls (95% CI [0.0%–0.3%], p=0.14). Regarding the incidence of what is traditionally termed “CIN” (defined as an increase of 25% or more within 96 hours of admission, but in this case regardless whether contrast was actually administered) 598 of 6,954 (8.6%, 95% CI [0.8%–9.3%]) receiving contrast met this criterion compared with 87 of 909 (9.6%, 95% CI [0.078–0.117]) patients not receiving contrast (p=0.32) (Table 2).

It is difficult to establish whether the contrast group was inherently a “sicker” group than the non-contrast controls, but it does not appear there were major differences. To be included, both groups were admitted to the hospital as inpatients. Mean length of stay for the contrast group was 5.3 days vs. 5.0 days in the non-contrast controls (p>0.75). Five hundred seventy-nine of 6,954 patients receiving contrast had any time in the intensive care unit (ICU) (8.3%) vs. 70 of 909 patients not receiving contrast (7.7%, p=0.39).

Of the 16 patients undergoing dialysis (all in the contrast group), it did appear that they all had significant medical conditions that might predispose to renal failure, even in the absence of contrast administration. Ten of the 16 patients underwent a surgical procedure (Table 3) including such major operations as aortic resection, hemicolectomy, coronary artery bypass grafting (CABG), and bowel resection. The six non-operative patients who underwent dialysis (Table 3) also appeared to have critical illnesses including sepsis, intubation, and gastrointestinal bleeding with shock. In no case did a patient without severe intercurrent illness who received contrast require dialysis. Despite these 16 isolated incidences (comprising less than 0.3% of all patients receiving IV contrast) there was no overall difference in dialysis between the contrast group and controls.

## DISCUSSION

The vast literature relating to CIN has focused almost exclusively on its detection and prevention as defined by a creatinine rise that varies from study to study; at least five different definitions have been used.^14^ In the current study our 8.6% incidence of CIN after contrast was squarely within the usual range. Although it is commonly noted that individual cases of severe renal failure, dialysis and death have occurred, it is uncertain how frequent such events are and there are no studies of ED populations comparing such patient-oriented outcomes in similar patients who did not receive contrast but did receive imaging. A recent article pertaining to the ED identified six patients (out of 633) with both study-defined CIN and serious adverse outcomes and concluded that “CIN was associated with severe renal failure and death from renal failure,” but all their patients had received contrast; there was no comparison group.^15^ The association between a rising creatinine and an adverse outcome (which included as “severe renal failure” a creatinine above 3.0) is not surprising. It is the unproven implication that the contrast administration was causally associated with the adverse outcomes that is of clinical relevance. Interestingly, the same authors, in a second paper, noted, “the precise contribution of the contrast load as the cause of the renal failure remains a matter of debate.”^16^ A recent ED study with a similar methodology to our own failed to demonstrate an increased risk of either CIN or adverse outcomes in the contrast-exposed group. In fact, the incidence of CIN itself was higher in the controls while mortality was the same in both groups.^17^ It appears that the temporal relationship between an increasing creatinine and receiving IV contrast has led to an assumption of causality that is not valid. As to the absolute incidence of CIN in those receiving contrast, a previous ED study in trauma patients (a younger and perhaps healthier cohort) reported an incidence of CIN of 5.1%.^18^ A huge meta-analysis comprising over 40 studies and almost 20,000 patients reported a similar point estimate of 6.4%.^19^

Although the proposition that intravenous contrast administration in patients with preserved renal function may be entirely free of renal toxicity may appear heretical to the emergency clinician, there is actually strong, if indirect, support for the idea in the radiology literature. Newhouse reported on an inpatient cohort of more than 30,000 patients followed for less than one week, none of whom received intravenous contrast. Remarkably, over half the patients had an elevation in creatinine of greater than 25%. Further, the elevation was even more likely in patients with the best renal function at baseline.^20^

## LIMITATIONS

Our study’s most serious limitation is that the contrast and control groups are undoubtedly dissimilar in ways that are not captured by the parameters we measured, particularly age, gender and diabetic status. Although it might appear that since abdominal CTs without contrast are much more likely to be used in patients where less serious disease is suspected (for example, those with renal colic, which would make our results even more remarkable), it may be that patients with a creatinine less than 1.6mg/dL, being scanned without contrast, who are then admitted represent a subgroup of particularly ill patients, although this was not evident in our analysis of length of stay or ICU admission. Similarly, those receiving a chest CT without contrast (perhaps pneumonia or cancer) are not obviously a more or less morbid group than those who do receive contrast, which would include, for example, all those in whom pulmonary embolism is suspected.

A second limitation is that we compared the contrast and control groups for only a limited number of variables that seemed most likely to be surrogate markers for a trait that would predispose to more (or fewer) instances of creatinine rise and adverse clinical outcomes. Our finding, that these characteristics, (age, gender, and diabetic status), were not different is consistent that of Sinert et al.^17^ who looked at many other factors as well, including race, insurance status, estimated creatinine clearance, lactate, bicarbonate, HIV, and sickle cell disease. They, too, found no explanation for the similarity in creatinine rise between those receiving contrast for CT and controls. As our controls, unlike theirs, all received a CT during hospital admission, their conclusion that their findings “further bring into question the current definition of contrast-induced acute kidney injury to differentiate the outcomes of contrast-exposed and contrast-unexposed patients” are confirmed and extended by our current, much larger study. Finally, a potential weakness, that the investigator compiling the dataset was aware of the study hypothesis, is unlikely to have had any effect. All data came from the ED and inpatient electronic medical records systems (EmStat® and Prism®). No charts were retrieved and no data were abstracted from chart review; no judgment was employed in determining eligibility. We included in the analysis all patients meeting our simple inclusion criteria (admission, abdominal or chest CT and two creatinine determinations within 96 hours of admission).

## CONCLUSION

A rise in the serum creatinine of 25%, usually used to define contrast-induced nephropathy, is equally common in patients admitted from the ED who received chest or abdominal CTs whether or not they received IV contrast. The important patient-oriented outcomes of death and dialysis were also not significantly more frequent in such patients receiving IV contrast than in those receiving no contrast at all. There do not appear to be demographic or clinical characteristics in either the contrast or non-contrast groups that correlate with an elevation in serum creatinine (referred to as CIN in those receiving contrast). The likelihood of serious clinical outcomes (death and dialysis) after abdominal or chest CT is also not significantly different in those two groups. As contrast CTs in published ED studies have been limited to patients with relatively preserved renal function these reassuring results should not be extrapolated to patients with significant renal compromise, a subset of the ED population in which further investigation is clearly warranted.

## Figures and Tables

**Table 1:** Patient characteristics.

Characteristics of patient	CT with IV Contrast=6954	CT without IV Contrast=909	P-value
Age	54 +/− 19.4	54 +/− 18.1	non-significant
Gender			
Male	3964 (57%)	482 (53%)	0.70
Female	2990 (43%)	427 (47%)	0.67
Diabetes	1207 (17.4%)	179 (19.7%)	0.077
LOS (days)	5.3	5.0	0.75
ICU (# percent)	579 (8.3%)	70 (7.7%)	0.39

*ICU*, intensive care unit; *LOS*, length of stay; *CT*, computed tomography; *IV*, intravenous

**Table 2:** Outcomes in the group receiving intravenous (IV) contrast vs. those not receiving IV contrast.

Outcomes	CT with IV Contrast=6954	CT without IV Contrast (control)=909	P-value
Serum creatinine increase by 25%	598 (8.6%)	87 (9.6%)	0.32
Dialysis	16 (0.23%)	0 (0%)	0.14
Death	106 (1.5%)	11 (1.25%)	0.24

*CT*, computed tomography

**Table 3:** Medical and surgical cases associated with inpatient dialysis.

Surgical Conditions Associated with Dialysis (10 cases)	Medical Conditions Associated with Dialysis (6 cases)
Aortic resection and replacement CABG Laparotomy Hemicolectomy (two cases) Lysis of adhesions Open lung biopsy Small bowel resection Radical pancreaticoduodenectomy Thoracic vessel resection and replacement	Diabetic with UTI Pancreatitis and HIV GI hemorrhage requiring intubation (had bleeding scan) Sepsis requiring intubation Pancreatitis requiring intubation Pneumonia requiring intubation and lung biopsy

*HIV*, human immunodeficiency virus; *CABG,* coronary artery bypass graft; *UTI*, urinary tract infection; *GI*, gastrointestinal
